# Early detection of radiation-induced lung damage with X-ray dark-field radiography in mice

**DOI:** 10.1007/s00330-020-07459-4

**Published:** 2020-11-19

**Authors:** Rico Burkhardt, Thomas Gora, Alexander A. Fingerle, Andreas P. Sauter, Felix Meurer, Stephan Umkehrer, Maximilian von Teuffenbach, Severin Kampfer, Daniela Schilling, Annette Feuchtinger, Axel K. Walch, Ernst Rummeny, Stephanie E. Combs, Thomas E. Schmid, Franz Pfeiffer, Jan J. Wilkens, Julia Herzen

**Affiliations:** 1 Department of Radiation Oncology, Technical University of Munich, School of Medicine and Klinikum rechts der Isar, Munich, Germany; 2 Institute of Radiation Medicine (IRM), Helmholtz Zentrum München, Neuherberg, Germany; 3 Physics Department, Technical University of Munich, Garching, Germany; 4 Department of Diagnostic and Interventional Radiology, Technical University of Munich, School of Medicine and Klinikum rechts der Isar, Munich, Germany; 5 Chair of Biomedical Physics, Technical University of Munich, Garching, Germany; 6 Munich School of BioEngineering (MSB), Technical University of Munich, Garching, Germany; 7 Abteilung Analytische Pathologie, Helmholtz Zentrum München, Neuherberg, Germany; 8 Deutsches Konsortium für Translationale Krebsforschung (DKTK), Partner Site Munich, Munich, Germany

**Keywords:** Radiography, X-rays, Mice, Lung, Sensitivity and specificity

## Abstract

**Objective:**

Assessing the advantage of x-ray dark-field contrast over x-ray transmission contrast in radiography for the detection of developing radiation-induced lung damage in mice.

**Methods:**

Two groups of female C57BL/6 mice (irradiated and control) were imaged obtaining both contrasts monthly for 28 weeks post irradiation. Six mice received 20 Gy of irradiation to the entire right lung sparing the left lung. The control group of six mice was not irradiated. A total of 88 radiographs of both contrasts were evaluated for both groups based on average values for two regions of interest, covering (irradiated) right lung and healthy left lung. The ratio of these average values, R, was distinguished between healthy and damaged lungs for both contrasts. The time-point when deviations of R from healthy lung exceeded 3σ was determined and compared among contrasts. The Wilcoxon-Mann-Whitney test was used to test against the null hypothesis that there is no difference between both groups. A selection of 32 radiographs was assessed by radiologists. Sensitivity and specificity were determined in order to compare the diagnostic potential of both contrasts. Inter-reader and intra-reader accuracy were rated with Cohen’s kappa.

**Results:**

Radiation-induced morphological changes of lung tissue caused deviations from the control group that were measured on average 10 weeks earlier with x-ray dark-field contrast than with x-ray transmission contrast. Sensitivity, specificity, and accuracy doubled using dark-field radiography.

**Conclusion:**

X-ray dark-field radiography detects morphological changes of lung tissue associated with radiation-induced damage earlier than transmission radiography in a pre-clinical mouse model.

**Key Points:**

*• Significant deviations from healthy lung due to irradiation were measured after 16 weeks with x-ray dark-field radiography (p = 0.004).*

*• Significant deviations occur on average 10 weeks earlier for x-ray dark-field radiography in comparison to x-ray transmission radiography.*

*• Sensitivity and specificity doubled when using x-ray dark-field radiography instead of x-ray transmission radiography.*

**Supplementary Information:**

The online version contains supplementary material available at 10.1007/s00330-020-07459-4.

## Introduction

Radiotherapy is a common treatment method for thoracic tumors that can come along with severe side effects for the lung, such as inflammation, fibrosis, or even cancer [1]. For optimal follow-up treatment, it is best when lung damages are detected as early as possible.

In clinical routine, changes in the lungs are detected using chest x-rays or computed tomography (CT). While the projectional data from chest x-rays provides limited spatial information and requires less dose, CT provides three-dimensional information of the lung at the cost of higher doses. In small-animal radiotherapy research, radiation-induced lung damage, emphysema [2–5], fibrosis [6–11], and imaging dose [12–16] have been investigated. Small-animal imaging with murine in vivo, micro-CT is used to measure the onset of lung fibrosis after irradiation [6, 17] or to study the inhibition of radiation-induced lung fibrosis [9]. Still, it is desirable to reduce the radiation dose required in small-animal micro-CT [13, 16] or to employ imaging techniques such as x-ray dark-field imaging [18, 19] which has been shown to provide sub-pixel information on the alveolar structure of lung tissue [20–22]. Radiographic x-ray dark-field imaging requires less dose than CT and has delivered promising results for the detection of inflammation [22], fibrosis [23], emphysema [24–27], and tumors [28] in mice. But the field still lacks data on the application of x-ray dark-field radiography in radiotherapeutic settings. Therefore, we investigated the combination of small-animal radiotherapy and radiography in a pre-clinical murine study determining a possible advantage of x-ray dark-field contrast over x-ray transmission contrast for the detection of developing radiation-induced lung damage.

## Materials and methods

### Setups and irradiation

Local irradiation of the lungs was performed with the Small Animal Research Platform (SARRP, Xstrahl Ltd) [29] and its treatment planning software MuriPlan. The planning CT was performed with 60-kVp x-rays filtered with 1-mm aluminum and irradiation was performed in a single fraction with 220-kVp x-rays filtered with 0.15-mm copper employing two opposing anterior and posterior oblique fields arranged to minimize the dose received by the heart and the spinal cord. The field size at the isocenter in the center of the right lung was 9 × 6 mm^2^. The distance to the source was 350 mm. Irradiation was realized by two matching subfields each using a fixed collimator of 9 × 3 mm^2^. The dose (absorbed dose to water, commissioned using a calibrated ionization chamber) to the isocenter was 20 Gy, at a dose rate of ~ 2 Gy/min.

Imaging was performed with a previously developed [30, 31] small-animal phase- and dark-field-contrast prototype system (SkyScan 1190, Bruker microCT). It is an experimental Talbot-Lau-interferometer utilizing grating interferometry to obtain transmission radiographs, as well as dark-field radiographs simultaneously [24, 30, 31]. While transmission radiography is based on the absorption of x-rays by the specimen, x-ray dark-field radiography is related to the scattering of x-rays [18, 30, 31]. It has been shown to be specifically suited for lung imaging providing sub-pixel information [20, 24, 28]. Dark-field radiographs differ from transmission radiographs as they depict the lung mostly without ribcage and spine. Because of the way the raw data is acquired, both types of radiographs are intrinsically co-registered. Acquisition was done with four-phase steps, 1.4-s exposure time per image, 37 kVp, 0.66 mA, and a visibility of 20%. The dose was measured with a cylindrical ionization chamber inserted in 7 mm of depth of a PMMA cylinder.

### Imaging study

The animal experiments for the presented imaging study were conducted in accordance with the German law for animal protection. The imaging study consisted of two groups of female C57BL/6 mice (Charles River Laboratories) which are sensitive to irradiation and the development of lung fibrosis [32]. Imaging was done immediately before irradiation and then monthly for 28 weeks. One mouse of the irradiated group and one mouse of the control group were used to histologically verify radiation-induced changes of lung tissue after 28 weeks.

The control group contained six mice at the beginning of the study. The number reduced to five due to premature death after 12 weeks. The irradiated group contained six mice which received 20-Gy x-ray irradiation on the entire right lung. The left lung was spared and used as a healthy reference. Both groups were imaged over the whole course of the study (88 radiographs in total, 3 excluded). Imaging was performed at eight points in time.

### Quantitative analysis

**Fig. 1:**
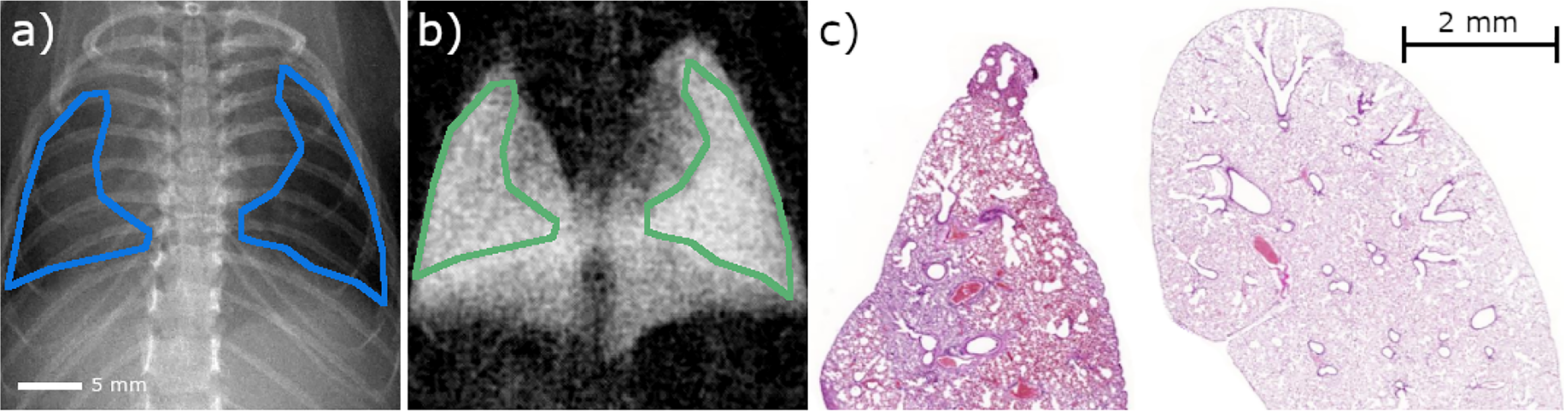
Positioning of regions of interest (ROI) in x-ray radiographs. Since absorption (**a**) and dark-field (**b**) radiographs are perfectly co-registered, the same mask can be used for extraction of pixel data. Shape and location of the ROIs were defined under the conditions to maximize the evaluated area while excluding the heart, bony peripheral regions of the ribcage and the spine. **c** Histologic sections (H&E staining) of a fibrotic lung (left) 20 weeks after irradiation with 20 Gy and a healthy lung (right)

All 88 radiographs were evaluated quantitatively for each imaging contrast. The analysis was based on the average values of regions of interest (ROI) in the radiographs. Size and location of the ROIs covered as much area of the lung as possible while excluding the spine and peripheral bony regions of the ribcage as well as the heart (Fig. 1). Since the transmission and dark-field radiographs are co-registered, the same ROIs were evaluated for both contrast types.

In each radiograph, the mean pixel value *m*_right_ of the right lung and the mean pixel value *m*_left_ of the left lung were determined. Since the left lung was never irradiated, the ratio R = *m*_right_/*m*_left_ represents either average transmission or average scattering normalized to healthy tissue. The ratio R should stay constant for the control group but in the irradiated group, it should change with the progress of the deterioration of the tissue of the right lung. This progress is expected to be individual for each mouse making a normal distribution unlikely in the irradiated group. Thus, *p* values were calculated using the Wilcoxon-Mann-Whitney test [33] with the null hypothesis that there is no difference between both groups in the ratio R for either contrast.

From the control group, the control region is derived. It is the average value of R over all points in time and all mice surrounded by a margin of 3σ (σ = standard deviation). The margin of 3σ was chosen so that all outliers of R for healthy mice are still within the control region. This control region is used to estimate at which point in time the deviation of R from the average value is larger than 3σ for individual mice. The point in time is obtained separately for transmission and dark-field contrast and then compared between both contrast types.

### Reader study

Three radiologists (A.S., F.M., A.F.) having 6, 4, and 12 years in clinical experience and 4, 2, and 9 years of experience with x-ray dark-field imaging assessed transmission and dark-field radiographs taken at three points in time (12, 20, and 28 weeks after irradiation) in two reads. This selection encompassed 32 radiographs for each contrast with 15 radiographs from the control group and 17 from the irradiated group. These radiographs were selected with two aims: to cover the space of time during which morphological changes become visible for all mice of the irradiated group and to include the endpoint of the study. Thus, the selected radiographs depict developing lung damage that eventually can lead to fibrosis. Before assessing the radiographs, the readers were informed that the lung of some of the mice was irradiated and then they received a training with radiographs of both contrast types for healthy and damaged lungs. This training material was taken from points in time not included in the reader study. The readers assessed the left and the right lung separately and classified them as either healthy or damaged. Thus, sensitivity and specificity are:

- Sensitivity: The mouse was irradiated and only the right lung was classified as damaged.
- Specificity: The mouse was not irradiated and both lungs were classified as healthy.

Furthermore, inter-reader and intra-reader accuracy were determined and rated with Cohen’s 𝜅 [34].

## Results

### Radiation dose and histology

The dose for the acquisition of all raw data images was 3 mGy. Histological evaluation was performed on formalin-fixed and hematoxylin eosin–stained lung tissue and can be seen in Fig. 1c. It showed a thickening of the alveolar walls in the affected area of the irradiated right lung, proving the occurrence of fibrosis.

### Quantitative analysis of transmission and dark-field radiographs

**Fig. 2:**
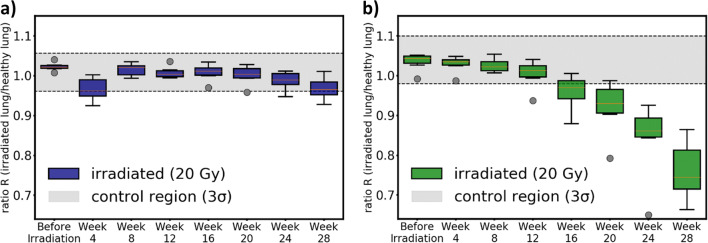
Results from the quantitative analysis. The boxes show the distributions of ratios R of the irradiated mice over a course of 28 weeks for transmission (**a**) and dark-field (**b**). The control region (grey area) was calculated from the non-irradiated control group. For transmission, the drop at week 4 stems from ointment that was used to treat skin inflammation. It increased absorption in the region of the right lung and therefore, the transmission decreased. Note that this effect can only be seen in transmission but not in dark-field. The decrease in dark-field begins between weeks 8 and 12 and in transmission, the decrease begins between weeks 20 and 24. In transmission, *p* values smaller than 0.01 are reached beyond 24 weeks while in dark-field, this value is reached between 12 and 16 weeks (see Table 2)

The development of the average value R over time is presented in Table 1 and Fig. 2. In the control group, the variation with time is less than 2% for transmission and less than 3% for dark-field radiography. This justifies the definition of the control region shown as a grey area in Fig. 2. In the irradiated group, decreasing R is observed for both contrasts. For x-ray transmission images, this decrease is due to the consolidation of lung tissue which increases the absorption of x-rays and decreases the measured transmission of the right lung. In x-ray dark-field images, the scarring of lung tissue causes a reduction of air-tissue interfaces decreasing x-ray scattering. Note that these changes in lung tissue are interrelated. After 28 weeks, the decrease in x-ray transmission contrast is below 6% and up to 26% in x-ray dark-field contrast. The prominent decrease for transmission in week 4 in Fig. 2a was due to ointment administered to treat skin inflammation. This inflammation was associated with the irradiation field and could be clearly seen due to loss of hair at the height of the lung. The ointment reduced the measured transmission but not the scattering of the right lung. In subsequent imaging, no ointment was required and therefore, the transmission is increased after week 4. For transmission, deviations larger than 3σ occurred only for three mice after 28 weeks. Figure 2b demonstrates that with dark-field, deviations larger than 3σ start occurring after 16 weeks. Table 2 shows that common significance levels (*p* = 0.05, 0.01) are reached earlier in dark-field than in transmission.

**Table 1:** Average values of the right-to-left-ratio R for transmission (T) and dark-field (DF) contrast over time. In the control group, the variation is below 3% over the course of the study for both contrasts. From these values, the control region is derived and it is represented by the grey area plotted in Fig. 2. In the irradiated group, the variation is less than 6% for transmission and up to 26% for dark-field

Group	Contrast type	Before irradiation	Week 4	Week 8	Week 12	Week 16	Week 20	Week 24	Week 28
Control	T	1.015	1.009	1.007	1.017	1.006	1.011	1.004	1.021
	DF	1.047	1.033	1.041	1.019	1.040	1.038	1.035	1.042
Irradiated (20 Gy)	T	1.023	0.966	1.016	1.008	1.008	1.001	0.987	0.968
	DF	1.035	1.029	1.026	1.004	0.958	0.919	0.841	0.760

**Table 2:** Calculated *p* values using the Wilcoxon-Mann-Whitney test for both imaging contrasts calculated relative to the control group at each point in time. The low *p* value for transmission after 4 weeks reflects the prominent deviation from the average value shown in Fig. 2. It is a consequence of ointment that was put onto the inflamed skin of the mice. One can see that common significance levels (*p* = 0.05, 0.01) are reached in week 16 for dark-field radiographs and in week 28 for transmission radiographs

Contrast type	Week 4	Week 8	Week 12	Week 16	Week 20	Week 24	Week 28
Transmission	0.010	0.811	0.158	0.676	0.463	0.158	0.004
Dark-Field	0.712	0.087	0.324	0.004	0.004	0.004	0.004

Regarding individual mice, deviations larger than 3σ were measured in dark-field before they were measured in transmission. The advantage in time was 10 weeks on average (min: 4 weeks, max: 16 weeks).

### Reader study

**Fig. 3:**
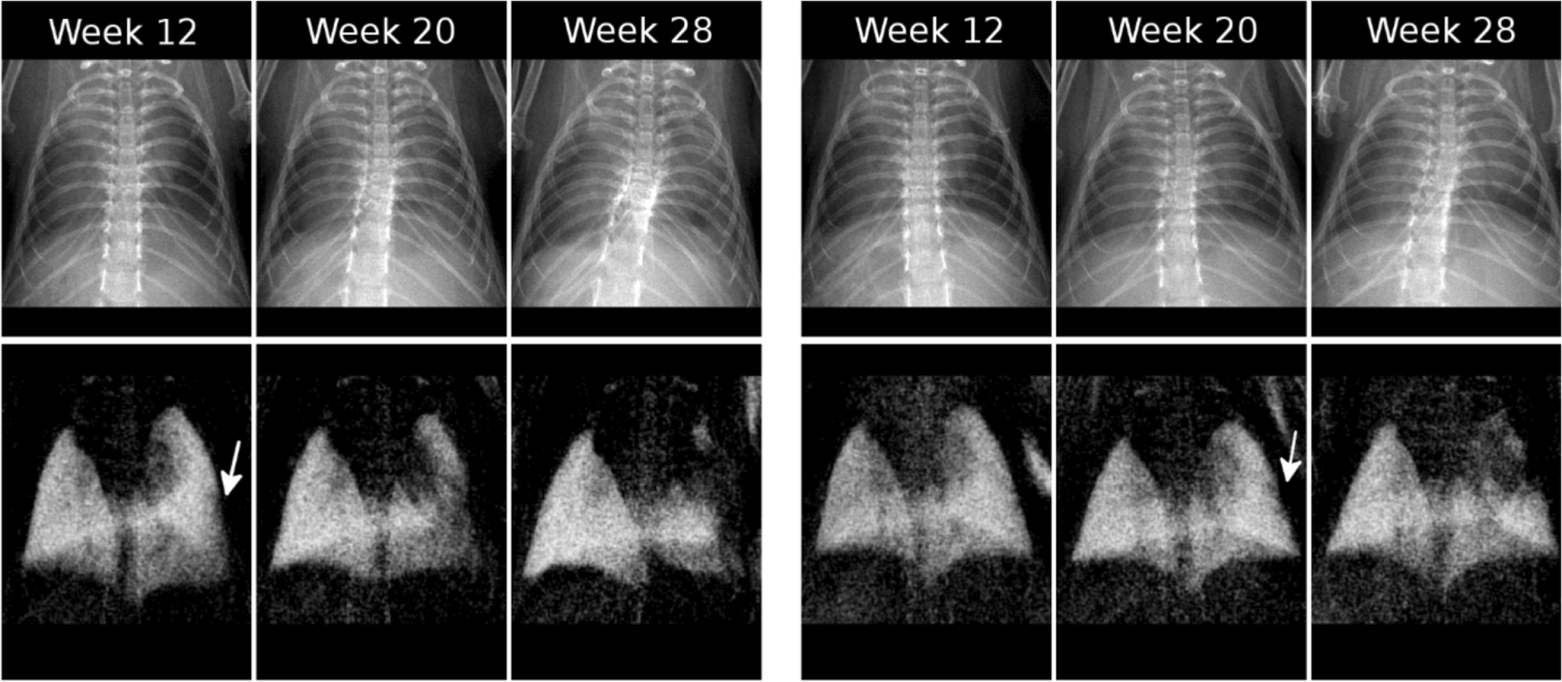
Selection of the radiographs that were assessed by radiologists (top: transmission, bottom: dark-field). Images from two different mice (left block and right block) are shown that were acquired 12, 20, and 28 weeks after irradiation of the entire right lung. The deterioration of lung tissue in the right lung can be seen clearly in the dark-field signal where it decreases over time. Also, in transmission images, lung deteriorations can be observed. More nuanced changes of the lung that occur at weeks 12 and 20 can only be observed in dark-field (white arrows)

A selection of radiographs assessed by the radiologists is presented in Fig. 3 (top: transmission, bottom: dark-field). Each of the two blocks represents one mouse and the images in one block cover both contrast types of radiographs acquired 12, 20, and 28 weeks after irradiation. The deterioration of the lung can be observed in both contrasts. First indications of lung damaged are marked with white arrows and occur after 12 or 20 weeks respectively.

**Fig. 4:**
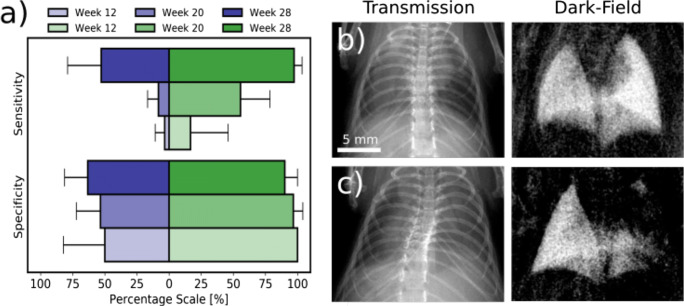
**a** Averaged sensitivity and specificity for transmission (blue) and dark-field (green) at weeks 12, 20, and 28. Specificity in dark-field is twice as high as in transmission. Sensitivity increases for both contrasts with time and is generally higher in dark-field in comparison to transmission. For transmission, most of the false classifications can be attributed to false classifications of the left lung which occurred for 24 of the 32 radiographs but with low frequency and almost no agreement between readers except for one case shown in subfigure **b**. Using dark-field in 3 of the 32 radiographs, the left lung was falsely classified as damaged. **b** Transmission and dark-field radiographs of a mouse from the control group. In transmission, 83% (5/6) agreement was found for the left lung being damaged while the right lung was classified as healthy. In dark-field, all readers agreed that the entire lung is healthy. **c** Transmission and dark-field radiographs of an irradiated mouse for which all readers entirely agreed in both contrasts that the right lung is damaged while the left is healthy

The results from the reader study are visualized in supplementary figure 1 and are summarized in Fig. 4 and Tables 3 and 4. Figure 4a shows the averaged sensitivity and specificity for transmission (blue) and dark-field (green) at weeks 12, 20, and 28. In dark-field, specificity is twice as high as in transmission with 100% in week 12 caused by uniform agreement across all six reads. Sensitivity increased with time for both contrasts and is generally higher for dark-field. Especially at weeks 20 and 28, damaged lungs were detected more frequently. In transmission, false classifications were eight times more often attributed to false classifications of the left lung (see supplementary figure 1). An illustrating example is shown in Fig. 4 b and c. Table 3 shows the pooled sensitivity and specificity for transmission and dark-field radiography. Average specificity was 95% in dark-field and 54% in transmission. Average sensitivity was 59% in dark-field and 23% in transmission. Note that in our study design, sensitivity is linked to progressing lung damage and thus, we should expect increasing sensitivity with time as seen in Fig. 4a. This fact then directly influences the pooled sensitivity shown in Table 3.

**Table 3:** Sensitivity and specificity for transmission and dark-field radiographs pooled over points in time given in percent. Tabulated are the results from the first and second read for each reader. Sensitivity and specificity are calculated for transmission (T) and dark-field (DF). On average, sensitivity and specificity are around twice as high in dark-field in comparison to transmission

	Sensitivity						Specificity					
	1^st^ reader		2^nd^ reader		3^rd^ reader		1^st^ reader		2^nd^ reader		3^rd^ reader	
	1^st^ read	2^nd^ read	1^st^ read	2^nd^ read	1^st^ read	2^nd^ read	1^st^ read	2^nd^ read	1^st^ read	2^nd^ read	1^st^ read	2^nd^ read
T	17.6	29.4	35.3	29.4	5.9	17.6	60.0	26.7	46.7	53.3	60.0	86.7
DF	64.7	53.0	41.2	47.1	52.9	94.1	100.0	93.1	86.7	93.1	100.0	100.0

**Table 4:** Accuracy of transmission and dark-field radiography obtained from the results of the reader study. When assessing dark-field radiographs, reader showed an accuracy above 50%. In transmission images, the accuracy was always below 50%. Furthermore, readers were more than twice as likely to repeat their assessment based on dark-field radiographs in comparison to transmission radiographs. Among readers, the accuracy is five to ten times higher in dark-field radiography. This is especially due to the high specificity (Table 3)

	Transmission contrast				Dark-field contrast			
	1^st^ reader (%)	2^nd^ reader (%)	3^rd^ reader (%)	Inter-reader (%)	1^st^ reader (%)	2^nd^ reader (%)	3^rd^ reader (%)	Inter-reader (%)
1^st^ read	37.5	40.6	28.1	12.5	81.3	62.5	75.0	62.5
2^nd^ read	28.1	31.3	50.0	6.3	71.9	68.8	96.9	65.6
Intra-reader	15.6	34.4	28.1	x	71.9	59.4	75.0	x

Table 4 shows single-reader, intra-reader, and inter-reader accuracy. With dark-field radiography, the readers tend more to repeat their assessment and also to come to the same assessment among readers. For two readers, 𝜅 = 0.21 for transmission and 𝜅 = 0.66 for dark-field. For three readers, 𝜅 = 0.08 for transmission and 𝜅 = 0.64 for dark-field.

## Discussion

We carried out a radiographic murine imaging study focused on the early detection of developing radiation-induced lung damage comparing the detection capability of x-ray dark-field contrast to x-ray transmission contrast. Two groups of mice were imaged monthly for 28 weeks: irradiated with 20 Gy and a control group. Both were evaluated quantitatively with an ROI-based analysis and in a reader study. Quantitative analysis showed that using dark-field contrast, significant deviations from healthy lung tissue could be measured between 16 and 20 weeks post irradiation and 10 weeks later with transmission contrast. These results were accompanied by doubling of sensitivity and specificity for dark-field in a reader study. Overall specificity was between 85 and 100% at 12, 20, and 28 weeks after irradiation using dark-field. Sensitivity at these points in time was always higher using dark-field and increased for both contrasts with progressing lung damage. Also, inter-reader and intra-reader accuracy were higher using dark-field. Overall, dark-field contrast performed more reliably for the detection of radiation-induced lung damage in murine specimen.

The murine model for irradiation-induced lung damage is representative of the anticipated pulmonary response in the human body [35]. In man, the absorption of ionizing radiation causes immediate biochemical, subcellular, and cellular damage but the morphological expression occurs delayed [1]. The rate of change depends on the dose and the irradiated volume with doses above 8 Gy leading to lung fibrosis 6 months after irradiation [1]. In mice, susceptibility to irradiation is strain-dependent [32] and doses above 12 Gy are required so that significant amounts of collagen could be found after 9 months which is used as an indicator for fibrosis [1]. In [36] is shown that in male LAF 1 mice, lung fibrosis might occur 20 weeks after irradiation for single doses between 12 and 15 Gy delivered to the entire body. Therefore, it depends on the point in time which kind of radiation-induced changes cause measurable deviations from healthy tissue. In our study, radiation-induced lung fibrosis was histologically confirmed 28 weeks post irradiation for female C57BL/6 mice. But for the previous points in time, we can only claim to have measured radiation-induced changes to lung tissue. These radiation-induced changes alter the alveolar structure influencing the absorption and scattering of x-rays. The advantage of x-ray dark-field radiography is explained by its ability to quantify the scattering of x-rays by the alveolar structure of lung tissue [21–23]. It has been shown to be sensitive to both enlarged and reduced alveoli [23, 26, 28]. The latter is related to scarring of the lung tissue as a consequence of radiation-induced damage typical for lung fibrosis [1, 37]. The scarring reduces air-tissue interfaces and thus scattering of x-rays. This scarring also consolidates lung tissue increasing absorption of x-rays measurable in transmission radiography and micro-CT. In small-animal imaging, only the latter has been employed so far for the detection of radiation-induced lung damages in male C57BL/6 mice [6, 7, 9, 10]. The reported points in time at which significant deviations from healthy tissue occur depend on the applied method [4, 6, 7, 17]. In [6], deviating HU values were measured after 1 and 4 days when the entire lung was irradiated with 20 Gy. In [7], doses of 20 Gy delivered to the whole thorax led to measurable deviations in HU 12 weeks after irradiation. And in [17], doses between 4 and 20 Gy were delivered to ~ 15% of the total lung volume leading to measurable deviations in HU after 10 weeks in male C57BL/6 mice. Visual confirmation of lung damage using micro-CT should be expected beyond 30 weeks post irradiation [2, 4–6, 28, 37]. Thus, it can be inferred that quantitative deviation does not have to match with visual confirmation. Our study showed that using dark-field radiography quantitative deviation and visual conformation occurred on time scales comparable to micro-CT. In previous x-ray dark-field radiography studies, the entire lung was affected by a disease in mice of either C57BL/6N or 129S/Sv-Kras strain [23, 25, 28], but in our study, the left lung was spared and could therefore be used as healthy reference in every radiograph. This method provided the possibility to quantify the advantage in time gained by x-ray dark-field radiography over transmission radiography. In terms of dose, 200 to 1000 mGy are given in micro-CT [5, 10, 12, 13, 17] while dark-field radiography requires less than 10 mGy [25, 28] like in our study. Although typical doses in micro-CT do not lead to increased radiotoxicity in C57BL/6 mice when delivered weekly [13, 16], higher doses were reported to cause life-shortening for ddY/SLC mice [15, 32].

Limitations of our study concern the imaging technique and the transferability to man. Although our results indicate an advantage of x-ray dark-field radiography, they are still based on a murine imaging study. Radiologists, however, are experts on human anatomy where the heart is found more caudal than in mice influencing the appearance of the upper right lung lobe. Furthermore, the acquired radiographs represent averages over several breathing cycles. Also, clinical radiographs provide more detail than radiographs of mice. For dark-field radiography, this deviation from an expected image is less pronounced because it is not part of clinical routine. Furthermore, evaluating sensitivity and specificity is not straightforward when dealing with a progressing disease because it is not clear at which point in time an irradiated lung is to be considered sick. Thus, there is a difference between the groups control and irradiated, and the groups healthy and sick. For example, if only lungs with deviations larger than 3σ would be considered sick, then the composition of the groups would change. As a consequence, sensitivity would then raise to up to 75% for transmission and up to 90% for dark-field. But for transmission radiography, the sick group would then mostly contain radiographs from the later points in time stages of the disease. Thus, nominal values would raise coming at the cost of maybe classifying early stages of lung damage as healthy. Additionally, beam hardening can also reduce the dark-field signal but that was not considered in our study since its influence is not yet known. Understanding its influence is recommended for future studies.

Altogether, our studies showed that dark-field radiography is able to quantitatively detect the onset of radiation-induced lung damages earlier than transmission radiography. The results from the reader study support the claim that dark-field radiography might be suited for the detection of lung damages at early stages. Therefore, x-ray dark-field radiography might become clinically relevant in the future.

## Supplementary information

Figure 1: Visualization of the results from the reader study for transmission (top) and dark-field (bottom) with legends in the top left. The results are broken down into groups and then further into points in time. Each line represents a single mouse at the specified point in time. Top: For transmission radiographs the distribution of false classifications is skewed to the left lung. Bottom: False classifications in the control group and of the left lung are scarce. (PDF 515 kb)

## Abbreviations

CT: Computed tomography
DF: Dark-field
HU: Hounsfield unit
m: Mean pixel value
ROI: Region of Interest
T: Transmission
