# Supplementary material for: Early detection of radiation-induced lung damage with X-ray dark-field radiography in mice
Source: Eur Radiol. 2020 Nov 19;31(6):4175–83. doi: 10.1007/s00330-020-07459-4 (PMC8128748; doi:10.1007/s00330-020-07459-4)
Supplement: Supplementary file 1 — Visualization of the results from the reader study for transmission (top) and dark-field (bottom) with legends in the top left. The results are broken down into groups and then further into points in time. Each line represents a single mouse at the specified point in time. Top: For transmission radiographs the distribution of false classifications is skewed to the left lung. Bottom: False classifications in the control group and of the left lung are scarce. (PDF 515 kb) [file 330_2020_7459_MOESM1_ESM.pdf]

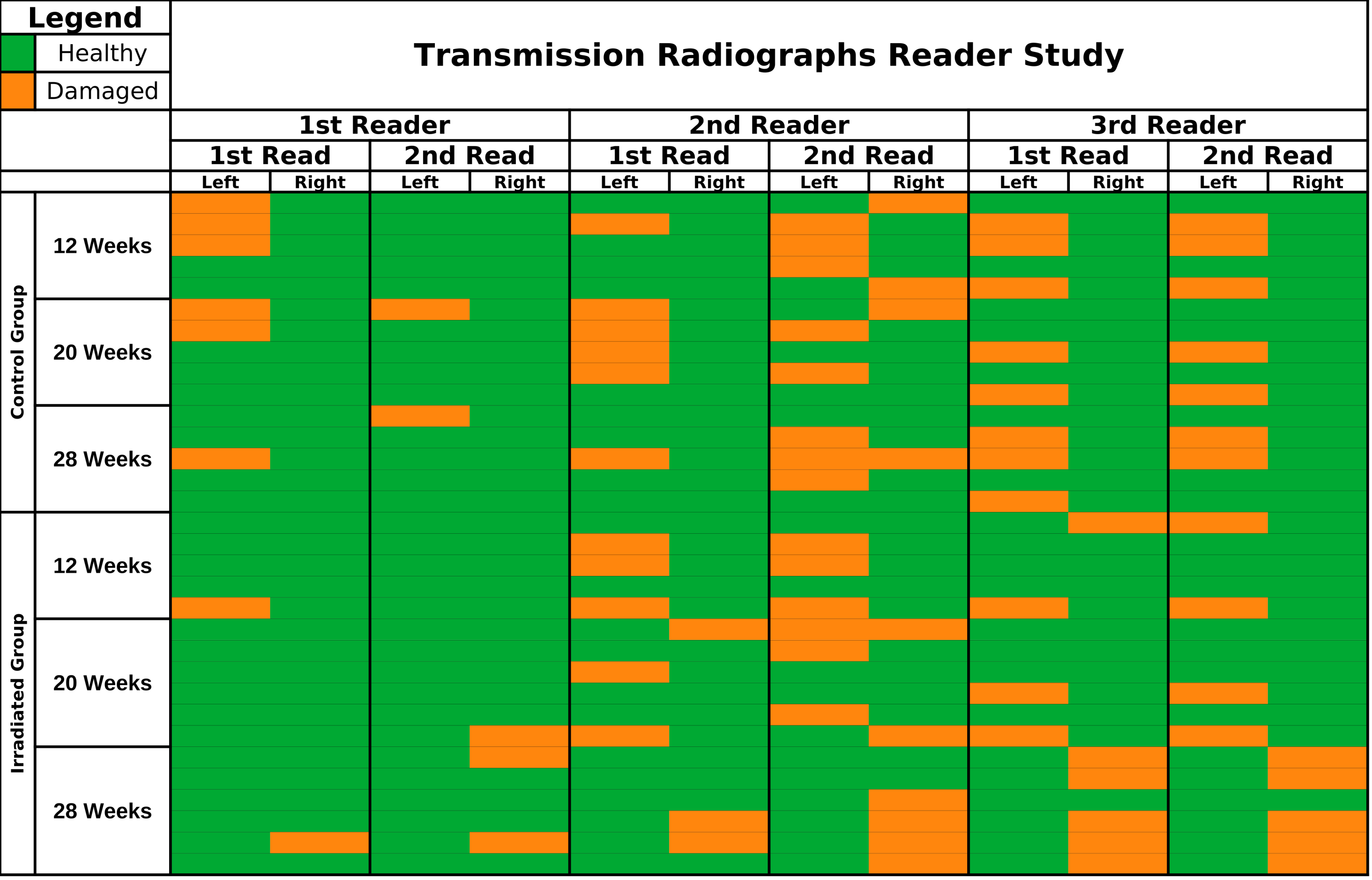
Legend
Healthy
Damaged
Transmission Radiographs Reader Study
1st Reader
2nd Reader
3rd Reader
1st Read
2nd Read
Left
Right
Control Group
Irradiated Group
12 Weeks
20 Weeks
28 Weeks

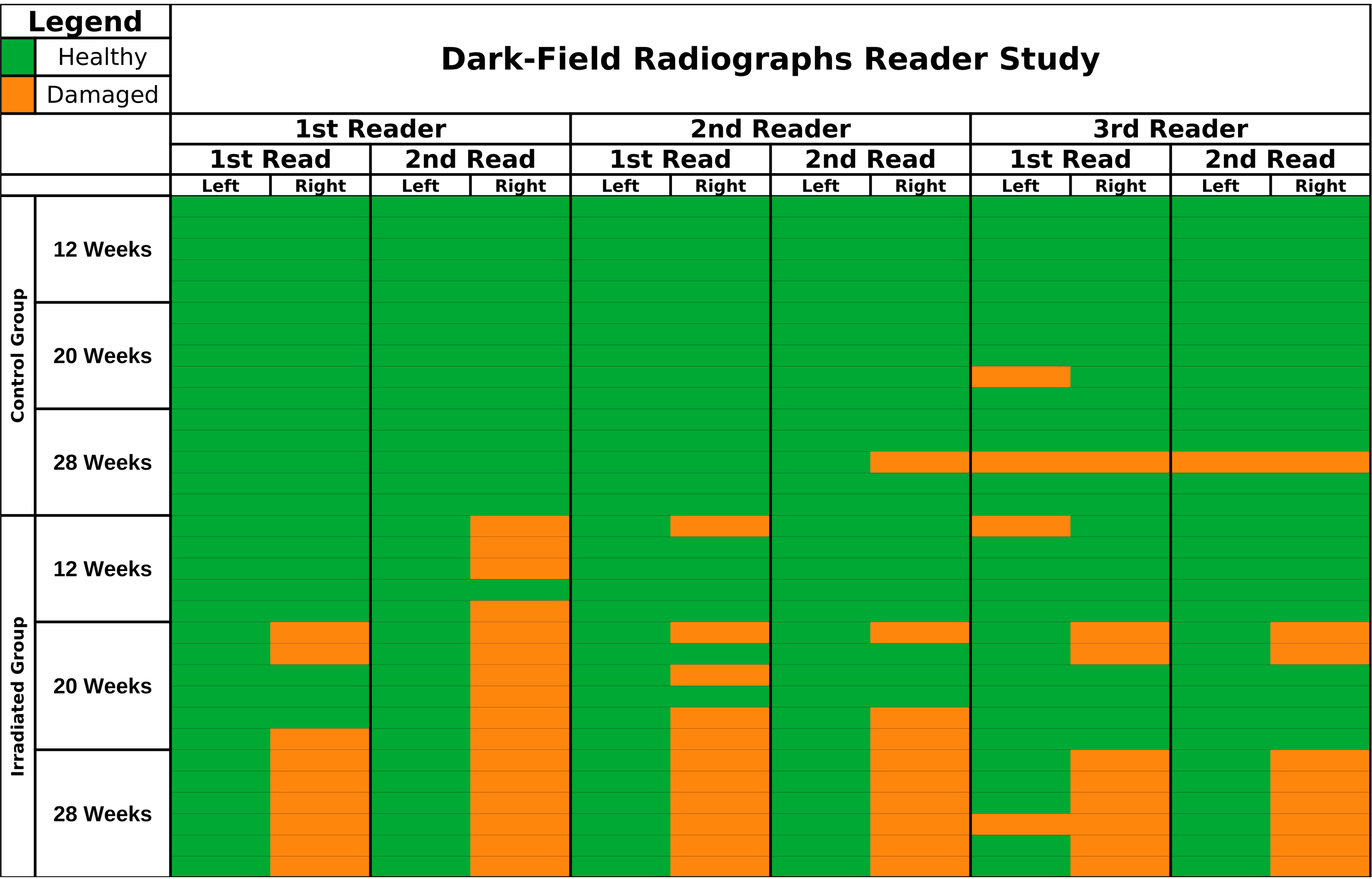
Legend
Healthy
Damaged
Dark-Field Radiographs Reader Study
1st Reader
2nd Reader
3rd Reader
1st Read
2nd Read
Left
Right
Control Group
Irradiated Group
12 Weeks
20 Weeks
28 Weeks
